# Seed set variation in wild *Clarkia* populations: teasing apart the effects of seasonal resource depletion, pollen quality, and pollen quantity

**DOI:** 10.1002/ece3.2372

**Published:** 2016-08-18

**Authors:** Alisa A. Hove, Susan J. Mazer, Christopher T. Ivey

**Affiliations:** ^1^ Biology DepartmentWarren Wilson CollegePO Box 9000AshevilleNC28815‐6217; ^2^ Ecology, Evolution, and Marine Biology DepartmentUniversity of CaliforniaSanta BarbaraCA93106; ^3^ Department of Biological SciencesCalifornia State University ChicoChicoCA95929‐0515

**Keywords:** Adaptation, *Clarkia unguiculata*, *Clarkia xantiana* ssp. *xantiana*, outcrossing, pollen limitation, pollination, resource limitation, self‐fertilization

## Abstract

In habitats where resource availability declines during the growing season*,* selection may favor early‐flowering individuals. Under such ephemerally favorable conditions, late‐blooming species (and individuals) may be particularly vulnerable to resource limitation of seed production. In California, a region prone to seasonal drought, members of the annual genus *Clarkia* are among the last to flower in the spring. We compared pollen limitation (PL) of seed set and outcrossing rates between early‐ and late‐flowering individuals in two mixed‐mating *Clarkia* taxa to detect whether flowering time is associated with changes in seed set due to resource depletion, PL, or increased selfing. In 2008–2010, we hand‐pollinated one flower on a total of 1855 individual plants either Early (near the onset of flowering) or Late (near the end of flowering) in the flowering season and compared seed set to adjacent, open‐pollinated flowers on the same stem. To assess the contribution of pollen quality to reproduction, we first (2008) used allozymes to estimate outcrossing rates of seeds produced by Early and Late open‐pollinated flowers. Second (2009), we conducted an anther‐removal experiment to estimate self‐pollen deposition. Seed set in *Clarkia unguiculata* was not pollen‐limited. *Clarkia xantiana* ssp*. xantiana* was pollen‐limited in 2008 and 2010, but not 2009. PL did not differ between Early and Late treatments. In both taxa, seed set of Early flowers was greater than Late flowers, but not due to PL in the latter. Reproduction was generally pollinator‐dependent. Most pollen deposition was xenogamous, and outcrossing rates were >0.7 – and similar between Early and Late periods. These results suggest that pollen receipt and pollen quality remain seasonally consistent. By contrast, the resources necessary to provision seeds decline, reducing the fitness benefits associated with resource allocation to ovules.

## Introduction

In outcrossing species, variation in seed set (the proportion of ovules that develop into seeds) within and among individuals may be determined by a number of factors, including the abundance and behavior of pollinators and the availability of resources necessary to provision seeds. While numerous studies have assessed the relative contribution of pollen and resource limitation to seed set (Haig and Westoby 1988; Campbell and Halama 1993; Casper and Niesenbaum 1993; Corbet 1998; Medrano et al. 2000; Ne'eman et al. 2006; Brookes et al. 2008; Groeneveld et al. 2010), fewer studies have examined the potential effects of intraseasonal variation in pollen limitation (PL) on seed set (but see Gross and Werner 1983; Dudash 1993; Forrest and Thomson 2008; Kameyama and Kudo 2009). Given that the identity of pollinator species and their abundances may be influenced by factors that vary seasonally (e.g., temperature, floral densities), many plant species may be predicted to experience PL at some point during flowering. To date, however, no general association between flowering phenology and PL has emerged. Elevated PL has been reported both early in the season (Thomson 2010) and late in the flowering season (Santandreu and Lloret 1999).

Identifying the factors that influence seed set is especially important for taxa that occupy habitats where resources decline predictably over the course of the growing season. In such habitats, the reproductive output of late‐blooming species (and individuals) may be particularly constrained by low‐resource availability, and selection may favor individuals that flower early (Hall and Willis 2006; Ivey and Carr 2012). Nonetheless, despite the increased risk of exposure to environmental stress, late flowering may be adaptive in such environments if it ameliorates the negative effects of early flowering, such as early‐season PL or reduced fitness from self‐pollination.

**Figure 1:**
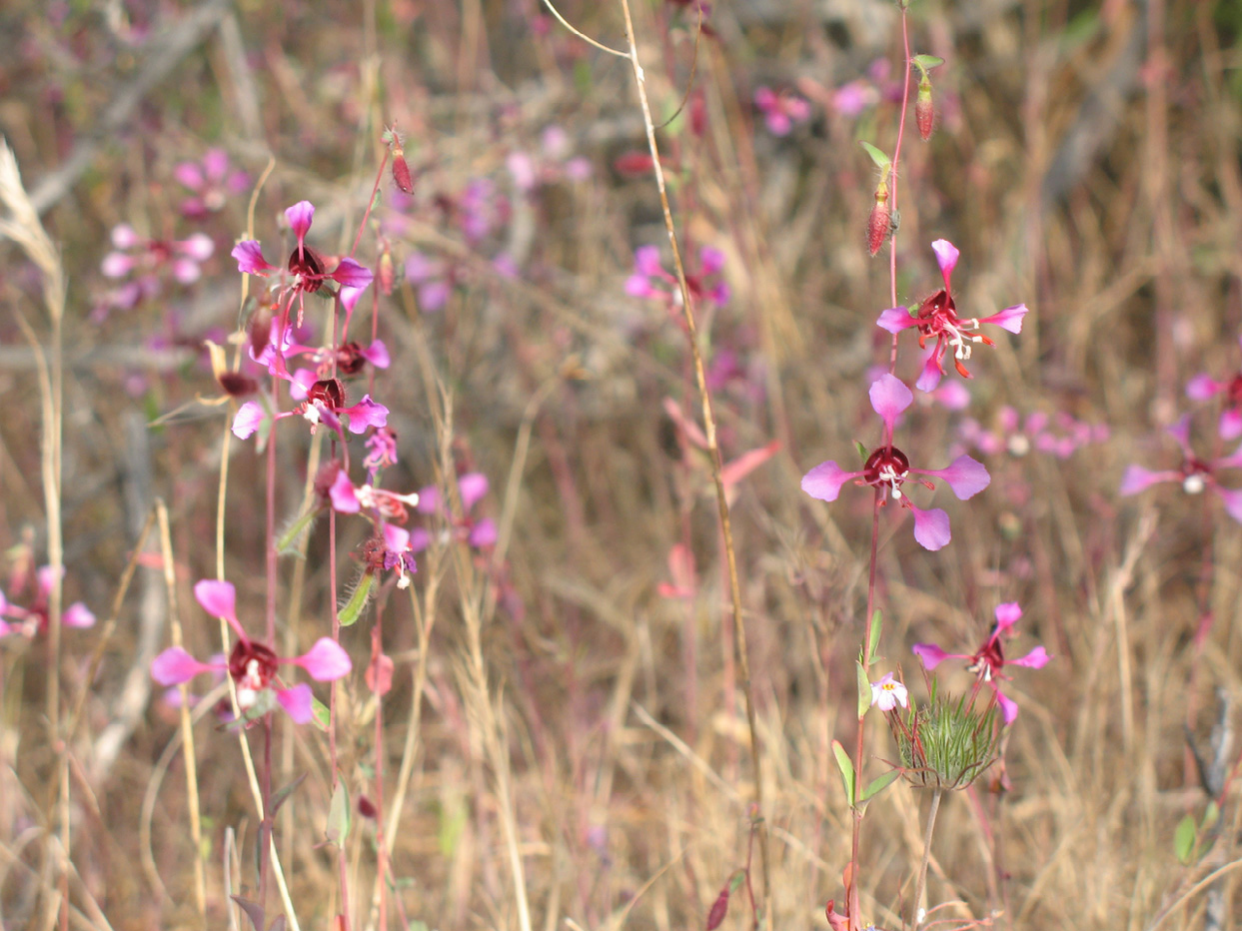
*Clarkia unguiculata* flowering in a population in the southern Sierra Nevada foothills. Photograph: Alisa Hove.

Predominantly outcrossing members of the annual self‐compatible wildflower genus *Clarkia* (Onagraceae, common name: farewell to spring, Fig. 1) are often among the last herbaceous species to flower in their plant communities. Because these taxa occupy habitats that regularly experience late‐spring drought, they provide an excellent opportunity to measure the relative contributions of temporal changes in pollen quantity, pollen quality, and resource availability on seed set. Two predominantly outcrossing *Clarkia* taxa, *Clarkia unguiculata* (hereafter “*unguiculata*”), and *Clarkia xantiana* ssp. *xantiana* (“*xantiana*”) occur at sites where water availability declines and temperatures increase throughout the growing season (Mazer et al. 2010). These taxa are the putative progenitors of sister taxa (*Clarkia exilis* [“*exilis*”] and *C. xantiana* ssp. *parviflora* [“*parviflora*”], respectively) that regularly self‐fertilize. Members of each pair of sister taxa differ in life history and physiology in a manner consistent with the hypothesis that their taxonomic divergence was driven in part by the timing of seasonal drought. Within each sister pair, field populations of the selfing taxon consistently exhibit traits associated with drought escape, including earlier flowering, shorter life cycles, and faster rates of photosynthesis than their outcrossing counterpart (Vasek and Sauer 1971; Eckhart and Geber 1999; Runions and Geber 2000; Mazer et al. 2010).

Pollen limitation, resource limitation, and the quality of pollen deposited on stigmas may collectively influence seed set in *unguiculata* and *xantiana* and may also influence their geographic ranges. Water availability has been proposed as a major determinant of the geographic range boundary of *xantiana* (Eckhart et al. 2010, 2011; Gould et al. 2014), and previous work has shown that at least some *xantiana* populations experience PL (Moeller 2004). Estimates of PL in natural *unguiculata* populations are lacking. Poor pollinator service, however, may have contributed to evolutionary divergence between *xantiana* and *parviflora,* and PL in marginal populations may limit range expansion at *xantiana's* range boundary (Moeller et al. 2012; Briscoe Runquist and Moeller 2014). These subspecies differ in both the composition of potential pollinator communities and the quality of pollination service; *xantiana* populations typically have higher pollinator visitation rates and harbor more *Clarkia*‐specializing bees than *parviflora* populations (Fausto et al. 2001; Moeller 2006). Although associations between pollen quality and seed set in these taxa are not well known, a negative effect of self‐pollination on individual seed mass or the fecundity of adult plants has been demonstrated in both taxa under greenhouse conditions*,* suggesting that the receipt of self‐pollen reduces offspring quality (Lowry 2007).

In habitats where mean daily temperatures and water stress increase throughout the growing season, one benefit of early flowering is that it reduces exposure to heat and drought stress. If abiotic conditions deteriorate during flowering, then flowers produced early in the season (whether within or among individual plants) should exhibit higher seed set than those produced late in the season. Indeed, declines in seed set between early and late flowers within individuals are routinely interpreted as evidence of resource limitation (Stephenson 1981; Brunet 1996; Forrest and Thomson 2010). Flowers produced early in the season, however, may experience higher PL if pollinators are less abundant or reliable prior to peak flowering. In this case, flowers produced early in the season would experience greater PL and potentially lower seed set than those produced late in the season. Here, we compared seed set and PL between early‐ and late‐flowering individuals in populations of *unguiculata* and *xantiana* to evaluate the importance of resource availability vs. pollen deposition in determining seasonal patterns of seed set. We also used two approaches to evaluate the degree to which pollen quality (estimated as the receipt of self‐pollen) changes temporally in both taxa. First, we used allozymes to estimate outcrossing rates from progeny arrays produced by early‐ vs. late‐flowering individuals in 2008. Second, we conducted an anther‐removal study to estimate the contributions of different sources of pollination (autogamy, geitonogamy, outcrossing) to pollen deposition in 2009.

This study addresses three central questions: (1) Is seed set in *unguiculata* and in *xantiana* consistently pollen‐limited among populations and years? PL has been well studied in *xantiana* (with evidence for context dependence; Moeller 2004), but this is the first study to measure PL in natural populations of *unguiculata*, providing a parallel test of its importance in populations where these species' distributions overlap. (2) Does the magnitude of PL change during the flowering season? (3) Does self‐fertilization differ between fruits derived from flowers produced early vs. late in the season, potentially contributing to temporal declines in seed set? Selfing estimates provide an indication of both the degree to which selfing provides reproductive assurance and the genetic quality of offspring, assuming nonzero inbreeding depression, even if seed set is not limited by insufficient pollination.

## Materials and Methods

### Study taxa and sites

*Clarkia unguiculata* Lindley and *C. xantiana* ssp. *xantiana* A. Gray are diploid annual taxa that occupy oak and pine woodlands, grasslands, and road cuts in the southern Sierra Nevada, CA. In the Lake Isabella region (Kern and Tulare Counties), both taxa may occur alone or co‐occur with their selfing sister taxa (*exilis* and *parviflora,* respectively) or with other *Clarkia* taxa (*Clarkia cylindrica*,* Clarkia rhomboidea*,* Clarkia speciosa*). In 2008–2010, we sampled three *unguiculata* populations (Live Oak, Mill Creek, and Stark Creek) and five *xantiana* populations (Borel Road, Camp 3, Lucas Creek, Sawmill 1.0, and Sawmill 3.3) in the Lake Isabella region. Populations were approximately similar in size (>3000 individuals), although the abundance of congeners and floral densities may also influence pollinator communities. We did not, however, collect floral density data or estimate population size at each site. See Table S1 for details regarding sampling locations, dates, and the presence of congeners at these sites.

Outcrossing *Clarkia* taxa are pollinated by specialist *Clarkia* bees and generalist bees (MacSwain et al. 1973; Moeller 2005, 2006). Both taxa are self‐compatible, but outcrossing is facilitated by strong spatial separation of anthers and stigmas within flowers (herkogamy) and the dehiscence of anthers prior to the onset of stigma receptivity (protandry) (Vasek 1958; Moore and Lewis 1965; Dudley et al. 2007). Mazer et al. (2010) provides detailed descriptions of both taxa.

### Testing for pollen limitation

To estimate the magnitude of early‐ and late‐season PL in *xantiana* and *unguiculata* in 2008–2010*,* we visited three populations per taxon twice per flowering season: at the onset of flowering (Early) and near the end of the flowering season after peak flowering (Late), with the exception of a single *xantiana* population (Camp 3) studied in 2009, which was sampled only Early. At a given site, Early and Late sampling dates were separated by a mean (SD) of 11.7 (5.6) days and the time interval between these sampling dates ranged from 5 to 23 days (Table S1). During each visit in 2008, we haphazardly selected 40 plants bearing at least one female‐phase flower (80 plants per population). Because of high levels of herbivory by caterpillars and browsing mammals in 2008, we sampled 200 plants per population in 2009–2010 (100 Early and 100 Late plants).

During each site visit, two flowers per plant (produced at successive nodes on the primary stem) were selected to receive one of two experimental treatments (one treatment per flower): (1). Pollen‐supplemented (S), or (2) Open‐pollinated (O), receiving no additional pollen. For the S treatment, pollen was applied to receptive stigmas with a blunted dissecting probe. To limit biparental inbreeding, supplemental pollen consisted of a mixture of pollen collected from 10 to 15 plants located at least 10 m from the focal plants and placed in a microcentrifuge tube no more than one hour prior to pollination. It was not feasible to measure PL at the whole‐plant level because the taxa studied here produce numerous flowers per plant and the study of multiple populations precluded frequent visits to each one. Previous comparisons of total seed production in *xantiana* following supplemental pollination of several flowers on the same plant vs. individual flowers, however, indicate that reallocation of resources to pollen‐augmented flowers does not result in elevated seed production or biased estimates of PL (Moeller et al. 2012).

In *unguiculata* and *xantiana*, seed set is influenced by a flower's position on the primary inflorescence; basal flowers on the inflorescence have higher seed set than distal flowers (see Fig. S1). Across individuals, Early flowers generally occurred at lower nodes than Late flowers, which could have influenced our ability to interpret seed set differences between the Early and Late sampling periods. In 2009 and 2010, to account for the effect of flower position on seed set, we recorded the position of each focal flower within the inflorescence and included this variable as a factor in our statistical models (below). The position of the first flower on the primary stem was designated as node “one.”

Mature fruits were collected at the end of each season; seed dispersal was prevented by applying a small drop of quick‐drying glue to the tips of capsules prior to dehiscence. Fruits typically contained fully developed seeds, partially filled ovules, and unfertilized ovules, which could be reliably identified with a dissecting microscope. Full seeds were large and plump, with dark, reticulate seed coats (Knies et al. 2004). Filled ovules were smaller and flatter than viable seeds and lacked well‐developed seed coats. Both viable seeds and filled ovules were easily distinguishable from the much smaller and pale‐colored unfertilized ovules. Seed set was quantified as the proportion of ovules within a fruit that developed into full seeds.

Among the individuals examined in 2008, mixed‐model ANOVAs were conducted to detect the factors influencing seed set. Pollination treatment (Supplemented or Open), timing of pollination (Early or Late), and pollination treatment × timing of pollination were treated as fixed effects, and population was treated as a random effect. The effects of population × treatment, population × timing of pollination, and population × treatment × timing of pollination were included in initial models as random effects, but they were removed from the final model because the 95% confidence intervals for the variance component estimates of these random effects included zero, indicating that they were not statistically significant. For all mixed‐model analyses, random effects were tested using restricted maximum likelihood and the Satterthwaite method was used to estimate degrees of freedom. Seed set data were arcsin square root transformed to meet the ANOVA's assumption of normality.

Among the individuals examined 2009 and 2010, we also used mixed‐model ANOVAs to evaluate variation in seed set, but these models also included the effects of flower position. Pollination treatment, timing of pollination, pollination treatment × timing of pollination, flower position, and timing of pollination × flower position were treated as fixed effects, and population was treated as a random effect. As above, all interactions were initially tested in a complete model, and nonsignificant terms were excluded from the final model.

In each year, the magnitude of PL was estimated using a PL Index. The PL Index for each individual plant was calculated as the difference between the mean seed set of the supplemental pollen treatment (S) and the mean seed set of the open‐pollinated treatment (O) standardized by the former (i.e., PL Index = (Supplemental − Open)/Supplemental). This analysis was restricted to plants from which both open and supplemental fruits were available for collection at the end of the season (i.e., intact fruits that were unconsumed by herbivores). Within each year and sample period, Wilcoxen sign‐rank tests were used to determine whether the PL Index differed significantly from zero. All analyses were performed using JMP Pro 11.2.0 (SAS Institute, Cary, NC, USA).

### Genetic estimates of selfing: outcrossing rates

The seeds within fruits produced by Early and Late open‐pollinated flowers in 2008 were used to compare outcrossing rates between plants sampled Early vs. Late in two populations of each taxon (*unguiculata*: Mill Creek and Stark Creek; *xantiana*: Borel Road and Camp 3). Seedlings from each open‐pollinated fruit ($\overset{¯}{x}=8.8$ seedlings/fruit) were assayed for up to seven allozyme loci (SHK, MDH, TPI, ALD, GOT, IDH, and DIA (Wendel and Weeden 1989). A mean of 19 families was available for analysis from each time period (Early and Late 2008).

Maximum likelihood estimates of multilocus outcrossing rates in Early‐ vs. Late‐maturing fruits were compared using Ritland's (2002) MLTR program. We estimated pollen and ovule allele frequencies separately and inferred maternal genotypes based on the progeny arrays. The Newton‐Raphson iterative procedure was used to estimate multilocus outcrossing rates (*t*_m_). Standard errors were estimated as the standard deviation of 1000 bootstrap replicates, with seed families as the unit of resampling. We used *z*‐tests to compare mating system parameters between Early and Late sampling periods.

### Ecological estimates of the potential for selfing: floral emasculation study

In flowers, there are three potential sources of pollen, all of which can contribute to seed production in *unguiculata* and *xantiana*. These sources are as follows: (1) outcross pollen delivered by animal pollinators (outcrossing), (2) self‐pollen delivered by within‐flower selfing (autogamy), and (3) self‐pollen from other flowers on the same plant (geitonogamy).

In 2009, to estimate the contribution of each pollen source to the pollen deposited on stigmas, 100 plants were haphazardly selected in three populations of each taxon (Table S2). The timing of each experiment in each population ranged from relatively early to relatively late in the flowering season. At each site, 25 clusters of four plants similar in both size and floral display (based on visual observation) were identified. On each plant in a cluster, a single focal flower was selected in which the anthers had fully dehisced, and the stigma was still unreceptive but appeared to be within 24 h of receptivity. All other flowers on the plant were considered to be “nonfocal flowers.” Each plant in a cluster was then randomly assigned to one of the four following treatments: (1) Focal flower intact, all nonfocal flowers and mature buds intact (II: intact/intact); (2) Focal flower intact, anthers removed from nonfocal flowers and mature buds (emasculated) (IE: intact/emasculated); (iii) Focal flower emasculated, nonfocal flowers and mature buds intact (EI: emasculated/intact); and (iv) Focal flower emasculated, nonfocal flowers and mature buds also emasculated (EE: emasculated/emasculated). Each treatment allowed the contributions of different pollen sources to pollen deposition to be distinguished (Table 1).

**Table 1:** The four experimental treatments applied in the 2009 floral emasculation study conducted in *xantiana* and *unguiculata* populations

Treatment	Treatment of focal flower	Treatment of nonfocal flowers and mature buds	Potential sources of pollen	*N*_*unguiculata*_	*N*_*xantiana*_
II	Intact	Intact	*O*,* A*,* G*	71	70
IE	Intact	Emasculated	*O*,* A*	53	59
EI	Emasculated	Intact	*O*,* G*	58	53
EE	Emasculated	Emasculated	*O*	68	66

Comparing the amount of pollen deposited on stigmas in each treatment provided an estimate of the relative contribution of each of the following mechanisms: outcrossing (*O*), geitonogamy (*G*), and autogamy (*A*) to pollen deposition. In predominantly outcrossing *Clarkia* taxa, flowers are protandrous; anthers shed pollen before stigmas become receptive. The duration of protandry, however, varies among individuals (Dudley et al. 2007). To avoid the possibility of autogamous self‐pollination, flowers in the EI and EE treatments were emasculated prior to stigma receptivity. Sample sizes for each treatment for each taxon are also provided.

To estimate the relative contribution of the three potential pollen sources [outcrossing (*O*), autogamy (*A*), and geitonogamy (*G*)], we compared mean pollen deposition between pairs of experimental treatments differing in one source of pollination. There were two pairs of experimental treatments that differed with respect to each pollen source. This allowed us to generate two estimates of each source's potential contribution to seed set (Table 2). For example, the difference between the amount of pollen received by the focal flowers in the IE and EE treatments provided an estimate of autogamous pollen deposition, as did the difference between the amount of pollen received by the focal flowers in the II and EI treatments.

**Table 2:** Treatment comparisons for the experiment estimating the contribution of different sources of pollination in 2009

Implications of treatment comparisons	
If (pollen load)_EE_ (*O*) < (pollen load)_IE_ (*O*,* A*), then autogamy contributes pollen	
If (pollen load)_EI_ (*O*,* G*) < (pollen load)_II_ (*O*,* A*,* G*), then autogamy contributes pollen	
If (pollen load)_EE_ (*O*) < (pollen load)_EI_ (*O*,* G*), then geitonogamy contributes pollen	
If (pollen load)_IE_ (*O*,* A*) < (pollen load)_II_ (*O*,* A*,* G*), then geitonogamy contributes pollen	
If (pollen load)_EE_ (*O*) = (pollen load)_II_ (*O*,* A*,* G*), then outcrossing contributes pollen	
Autogamous pollen deposition (*A*)	
*A*_1_ = (pollen load)_IE_ − (pollen load)_EE_	*A*_2_ = (pollen load)_II_ − (pollen load)_EI_
Geitonogamous pollen deposition (*G*)	
*G*_1_ = (pollen load)_EI_ − (pollen load)_EE_	*G*_2_ = (pollen load)_II_ − (pollen load)_IE_
Outcross pollen deposition (*O*)	
*O*_1_ = (pollen load)_II_ − (*A*_1_ + *G*_1_)	*O*_2_ = (pollen load)_II_ − (*A*_2_ + *G*_2_)

Pollen sources available to plants included in each treatment group (II, IE, EI, EE) are included in parentheses (*O *= outcrossing, *G *= geitonogamy, and *A *= autogamy). The experimental design provides two independent estimates of each pollination source's contribution to pollen deposition.

After 48–72 h, stigmas from all focal flowers were harvested and preserved in formalin acetic acid. While we aimed to collect 100 stigmas per site, our sample size was reduced because some stigmas fell from plants or were consumed by herbivores prior to collection. We successfully harvested a total of 250 *unguiculata* and 246 *xantiana* focal flowers (Table S2). Stigmas were treated with Alexander's Stain (Kearns and Inouye 1993), squashed between a coverslip and a glass slide, and observed with a dissection microscope. Each stigma's pollen load (i.e., number of pollen grains deposited) was recorded.

Mixed‐model ANOVAs were used to test for differences in pollen loads due to treatment (fixed effect), population (random effect), and the population × treatment interaction (random effect) in each taxon. To meet the ANOVA's assumption of normality, pollen load data were log_10_‐transformed prior to analysis.

## Results

### Pollen limitation

**Figure 2:**
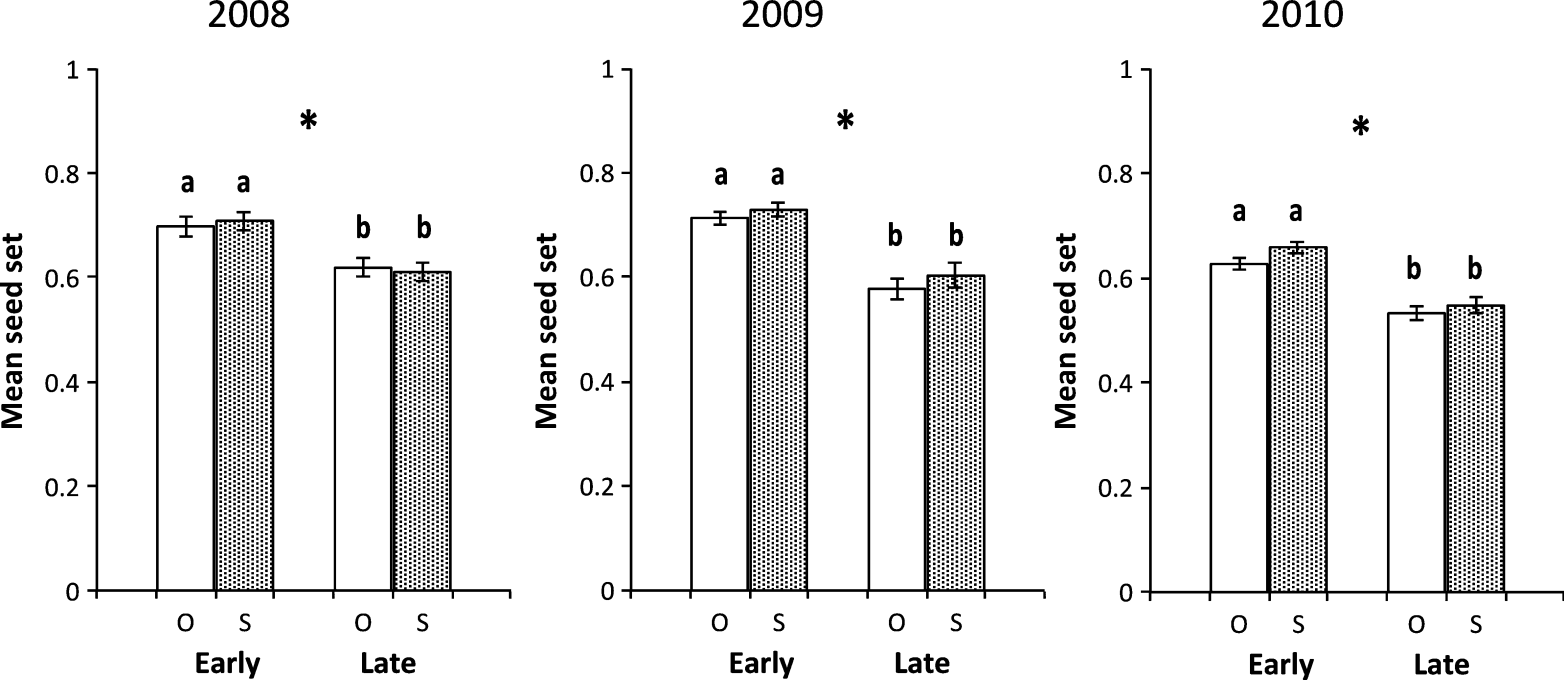
Mean seed set (±1 SE) in *unguiculata* in open‐pollinated (O, open bars) vs. pollen‐supplemented (S, stippled bars) flowers sampled Early and Late in 2008, 2009, and 2010. Asterisks (*) indicate years when Early and Late mean seed set differed statistically (mixed‐model ANOVA,* P *<* *0.05, Table 4). Within years and sampling periods, identical letters indicate similar seed set between pollination treatments (mixed‐model ANOVA,* P *>* *0.05, Table 4).

**Figure 3:**
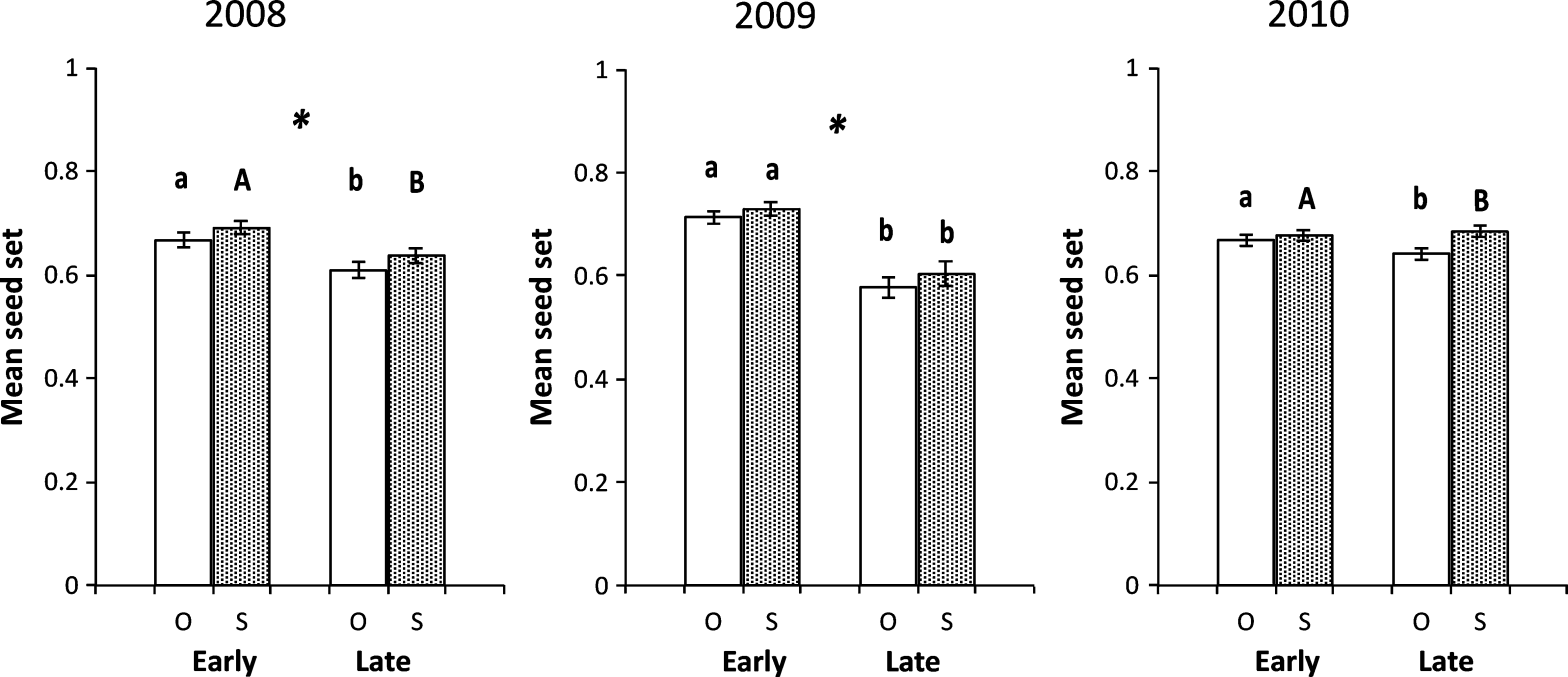
Mean seed set (±1 SE) in *C. xantiana* in open‐pollinated (O, open bars) vs. pollen‐supplemented (S, stippled bars) flowers sampled Early and Late in 2008, 2009, and 2010. Asterisks (*) indicate years in which Early and Late mean seed set differed statistically (mixed‐model ANOVA,* P *<* *0.05, Table 5). Within years and sampling periods, letters that differ in capitalization (e.g., A vs. a or B vs. b) indicate a significant difference between pollination treatments (mixed‐model ANOVA,* P *<* *0.05, Table 5).

We found little evidence of pollen‐limited seed set in *unguiculata* during the three years of this study. Fruits produced by pollen‐supplemented flowers consistently exhibited levels of seed set similar to their open‐pollinated counterparts (Table 3, Fig. 2). By contrast, seed set was pollen‐limited in *xantiana* populations. PL, however, was not consistent across years; we detected significant PL in 2008 and 2010, but not in 2009 (Table 4, Fig. 3). Within each year, PL did not change over time in *xantiana*; we found no evidence of a significant pollination treatment × pollination timing interaction (Table 4). Overall, estimates of PL were low (Table 5); the PL Index differed significantly from zero for two of the six sampling periods of the study (Early 2010 in *unguiculata* and Late 2010 in *xantiana*, Table 5).

**Table 3:** Summary of mixed‐model ANOVAs showing the effects of fixed factors and of population (random factor) on seed set in *unguiculata*

Year	Effect	df_N,D_	*F* ratio or variance component
2008	Timing of pollination	1, 296.5	**36.7822**
	Pollination treatment	1, 296	0.0263
	Timing of pollination × pollination treatment	1, 296	0.16
	Population		0.0074
2009	Timing of pollination	1, 464	**71.0646**
	Pollination treatment	1, 464	1.3021
	Flower node	1, 464.2	1.6343
	Timing of pollination × pollination treatment	1, 464	0.0181
	Timing of pollination × flower node	1, 464.5	0.4052
	Population		0.0080
2010	Timing of pollination	1, 835.8	**26.1865**
	Pollination treatment	1, 887.9	3.2576
	Flower node	1, 889.1	3.7542
	Timing of pollination × pollination treatment	1, 887.8	0.5065
	Timing of pollination × flower node	1, 601.6	**7.4029**
	Population		0.0002

*F* ratios are indicated for fixed effects. Variance components correspond to random population effects. Significant effects (for fixed effects: *P* < 0.05; for random effects, the 95% confidence interval of the variance component does not include zero) are indicated in bold.

**Table 4:** Summary of mixed‐model ANOVAs showing the effects of fixed factors and of population (random factor) on seed set in *xantiana*

Year	Effect	df_N,D_	*F* ratio or variance component
2008	Timing of pollination	1, 340	**12.3929**
	Pollination treatment	1, 338.9	**4.2057**
	Timing of pollination × pollination treatment	1, 339	0.1770
	Population		−0.00004
2009	Timing of pollination	1, 688.9	**119.0955**
	Pollination treatment	1, 712.1	0.7921
	Flower node	1, 711.5	**10.7608**
	Timing of pollination × pollination treatment	1, 712.2	0.2396
	Timing of pollination × flower node	1, 712.5	**4.8269**
	Population		0.0030
2010	Timing of pollination	1, 802.8	3.2673
	Pollination treatment	1, 801	**5.7083**
	Flower node	1, 801.6	**5.8855**
	Timing of Pollination × pollination treatment	1, 801	2.2294
	Timing of pollination × flower node	1, 801.1	0.6248
	Population		0.0055

*F* ratios are indicated for fixed effects. Variance components are associated with random population effects. Significant effects (for fixed effects: *P* < 0.05; for random effects, the 95% confidence interval of the variance component does not include zero) are indicated in bold.

**Table 5:** Summary of mean seed set (SE) and PL Indices of Early and Late open‐pollinated and pollen‐supplemented fruits for each year of the study

Taxon	Year	Timing	*n*	Mean seed set_open_ (SE)	Mean seed set_supp_ (SE)	PL index
*unguiculata*	2008	Early	65	0.693 (0.020)	0.705 (0.019)	0.018
		Late	76	0.612 (0.020)	0.622 (0.018)	0.016
	2009	Early	149	0.714 (0.014)	0.728 (0.013)	0.020
		Late	72	0.578 (0.017)	0.576 (0.027)	−0.004
	2010	Early	240	0.626 (0.013)	0.658 (0.011)	0.048*
		Late	182	0.540 (0.023)	0.559 (0.016)	0.034
*xantiana*	2008	Early	71	0.664 (0.010)	0.692 (0.013)	0.040
		Late	98	0.598 (0.023)	0.622 (0.017)	0.038
	2009	Early	245	0.691 (0.013)	0.700 (0.010)	0.013
		Late	167	0.329 (0.015)	0.331 (0.025)	0.007
	2010	Early	218	0.668 (0.011)	0.678 (0.011)	0.014
		Late	169	0.649 (0.012)	0.682 (0.011)	0.049*

The PL Index = (Seed set_supp_ − Seed set_open_)/Seed Set_supp_. PL indices that differed significantly from zero (Wilcoxen sign‐rank test, *P* < 0.05) are indicated with an asterisk (*).

### Seed set of Early‐ vs. Late‐blooming flowers

In both taxa, the timing of pollination influenced seed set in all or most years of this study. Seed set among Early‐blooming flowers was significantly higher than that of Late flowers in *unguiculata* in all 3 years. Elevated seed set in Early flowers was observed even when controlling position effects in 2009 and 2010 (Table 3, Fig. 2). In *xantiana*, Early‐blooming flowers had significantly higher seed set than Late‐blooming flowers in 2008 and 2009. In 2010, however, mean seed set did not differ between Early‐ and Late‐blooming flowers in this taxon (Table 4, Fig. 3).

### Outcrossing estimates in 2008

**Figure 4:**
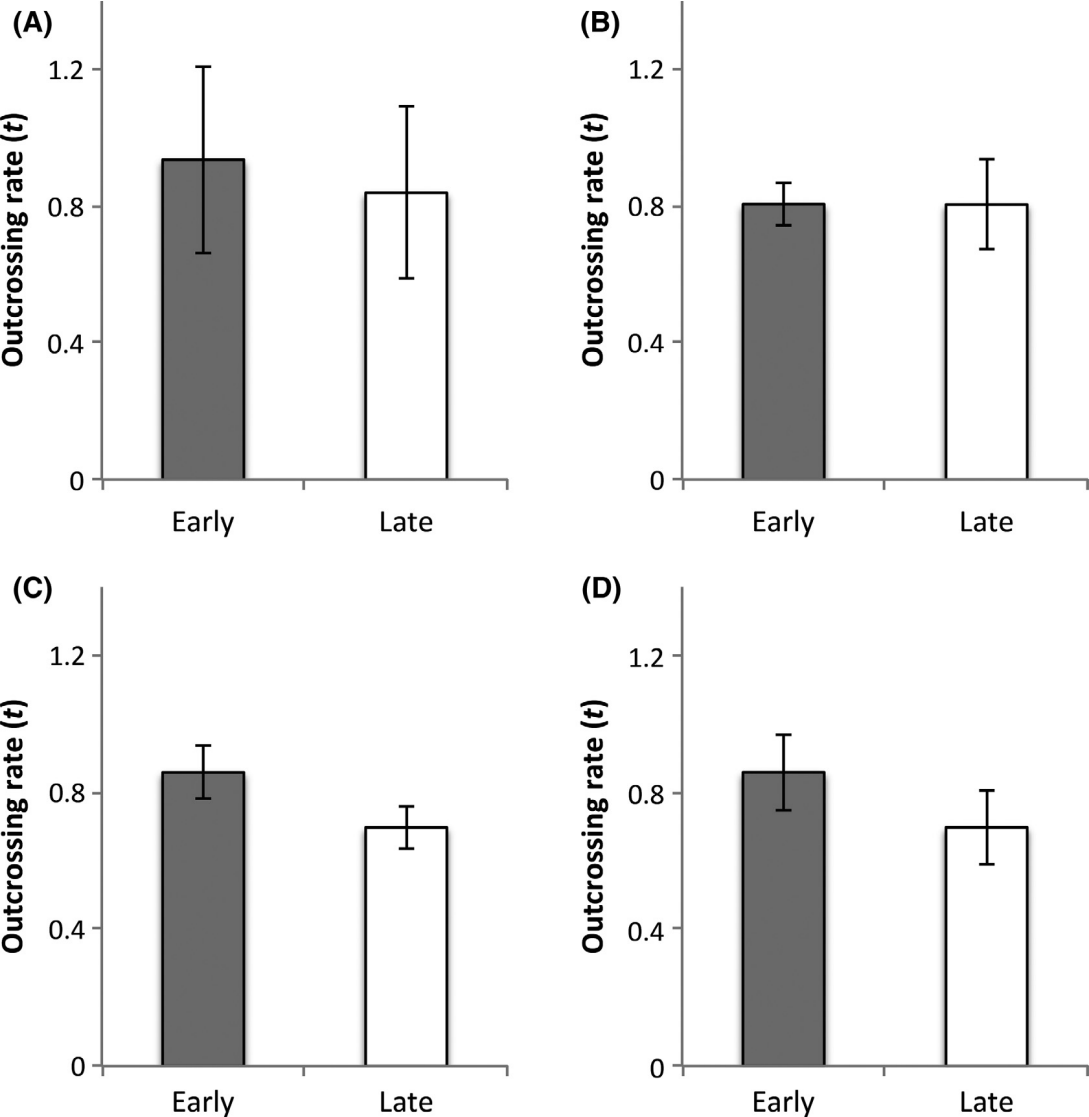
Multilocus outcrossing rates (*t*_m_) (±1 SE) for open‐pollinated Early (gray bars)‐ and Late‐blooming (white bars) flowers of from *unguiculata* populations (A: Mill Creek and B: Stark Creek) and x*antiana* populations (C: Borel Road and D: Camp 3) in 2008. Estimates were generated from allozyme variation in progeny arrays using MLTR (Ritland 2002).

Estimates of the multilocus outcrossing rate were high in both taxa (Fig. 4). In *unguiculata* populations, *t*_m_ was generally ≥0.80. Estimates of *t*_m_ in *xantiana* populations were slightly lower, ranging from 0.70 to 0.95. Early and Late outcrossing rates did not significantly differ in any population (Fig. 4).

### Sources of pollen deposition in 2009

**Figure 5:**
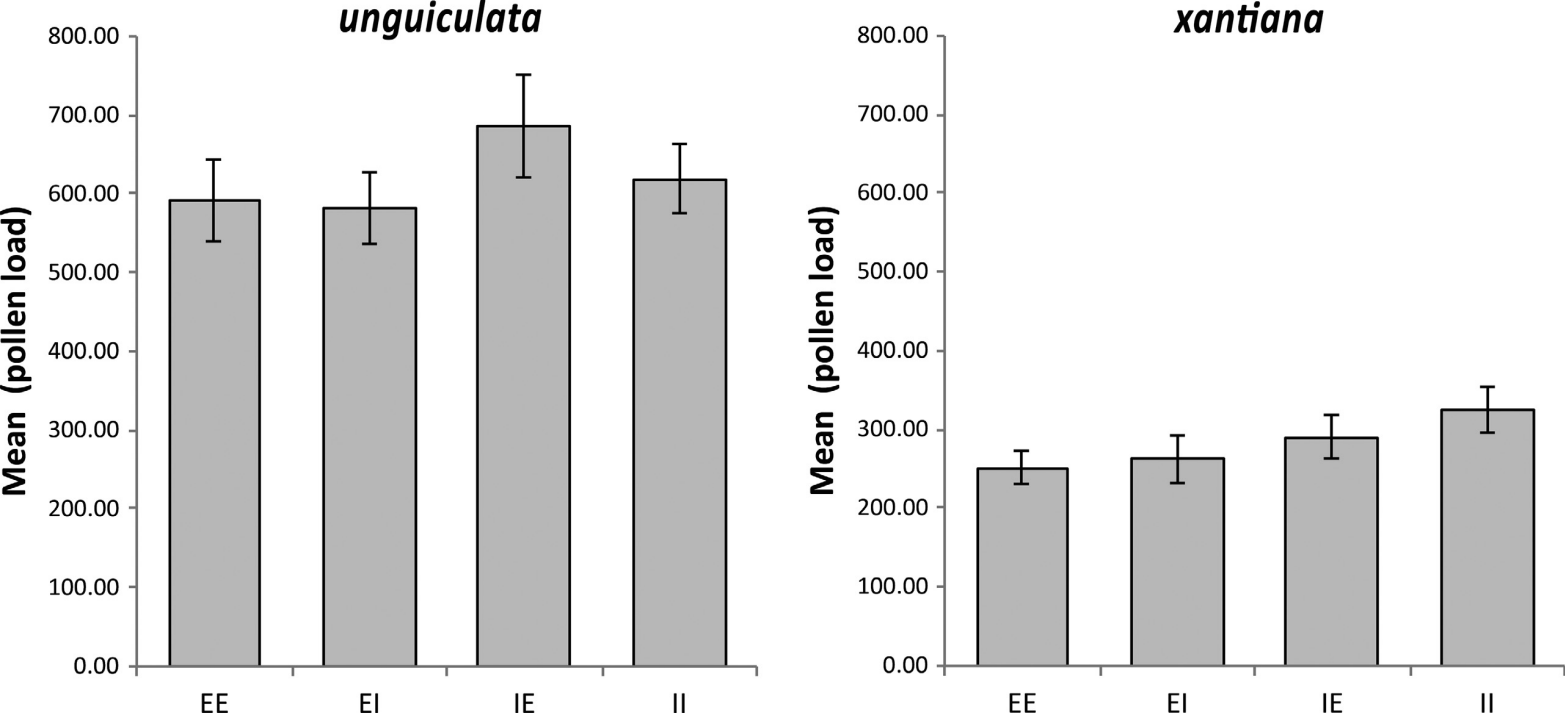
Mean pollen deposition (±1 SE) following each of the four experimental treatments imposed in *unguiculata* and *xantiana* populations in 2009. Possible sources of pollen deposition are indicated in parentheses below each treatment (*O *= outcrossing, *A *= autogamy, and *G *= geitonogamy).

Mean pollen deposition was similar among the four experimental treatment groups in both *unguiculata* and *xantiana* (Table 6, Fig. 5). Focal flowers in the EE treatment (all open flowers and mature buds emasculated) received as many pollen grains as focal flowers in the control treatment (II, all flowers intact), indicating that most of the pollen received by focal flowers of the II treatment originated from other plants. Estimates of outcross pollen deposition, O_1_ and O_2_ (Table 2)*,* indicated that cross‐pollination contributed 70–100% of total pollen deposited on II flowers in *unguiculata* populations. In *xantiana* populations, an estimated 82–100% of total pollen deposition on II flowers resulted from cross‐pollination.

**Table 6:** Summary of mixed‐model ANOVAs showing the effects of experimental treatment (fixed), population, and population × treatment (random factors) to sources of pollen deposition (autogamy, geitonogamy, xenogamy) in *unguiculata* and *xantiana* in 2009

Taxon	Effect	df_N,D_	*F* ratio or variance component
*unguiculata*	Treatment	2, 5.8	0.5121
	Population		0.0475
	Treatment × population		0.0029
*xantiana*	Treatment	3, 4.7	2.433
	Population		0.0560
	Treatment × population		−0.0006

None of the effects were statistically significant (for fixed effects: *P* < 0.05; for random effects, the 95% confidence interval of the variance component does not include zero).

Mean pollen loads did not differ significantly within either *unguiculata* or *xantiana* (Table 6). On average, pollen loads were higher in *unguiculata* ($\overset{¯}{x}=616.95$, SE = 25.79, *n* = 250) than in *xantiana* ($\overset{¯}{x}=283.92$, SE = 13.60, *n* = 246). Mean pollen loads observed in pooled populations of each taxon, however, greatly exceeded the mean number of ovules per flower, which is ~80–100 (Delesalle et al. 2008).

## Discussion

Temporal reductions in seed set can be intuitively explained as being due to resource limitation (Brunet 1996; Medrano et al. 2000; Vallius 2000). An alternative explanation, however, is that seasonal declines in seed production occur due to declines in pollinator availability, resulting in reductions in seed set due to the insufficient receipt of outcross pollen or due to the receipt of comparatively low‐quality self‐pollen.

The *Clarkia* populations in this region experience increasing mean daily temperatures and decreasing rainfall during flowering and fruit set (May–July; Mazer et al. 2010; Dudley et al. 2015). Consequently, it is reasonable to expect that the seasonal depletion of soil moisture during seed development would result in a temporal reduction in mean seed set among flowers produced early vs. late in the flowering periods of *Clarkia* populations in this region. As seasonal drought intensifies during flowering, however, it is also possible that pollinator service declines, contributing to temporal reductions in seed set due to increases in either PL or the proportion of self‐pollen deposited on stigmas.

To assess the independent contributions of PL, seasonal resource declines, and pollen quality to seed set per flower, we compared the seed set of pollen‐supplemented and open‐pollinated flowers at two points during the flowering season (Early and Late) over a three‐year period. Both *unguiculata* and *xantiana* are among the last herbaceous species to flower in their plant communities. Despite the rapid onset of seasonal drought at these sites, we found no evidence of seasonal changes in PL or pollen quality (estimated as the proportion of selfed seeds per fruit) between Early and Late flowers. In both taxa and in most years (with the exception of *xantiana* in 2010), however, we observed significant declines in seed set between the Early and Late plants. The number of seeds per fruit, as well as the total number of ovules per fruit, adjusted for node position, declined over time (along with seed set), consistent with the interpretation that resource availability and/or growing conditions declined over the course of the growing season.

On average, the proportion of ovules that matured into full seeds per fruit did not reach 100% in either taxon (Figs. 2, 3), even among pollen‐supplemented flowers blooming at the start of the flowering season. The factors preventing 100% seed set in these populations could not be identified in the current study. Further studies simultaneously manipulating water and pollen availability within and across years could help more fully to assess the roles of pollen and resource limitation in these taxa. For example, an experiment in which early‐ and late‐season water availability (through supplemental irrigation) *and* pollen deposition are manipulated in the field could help to determine whether seed set in these taxa is more consistently resource‐limited than pollen‐limited.

Patterns of PL differed between the taxa throughout the three‐year study. Seed set of *xantiana* was pollen‐limited in two of the three years of this study, a phenomenon that was observed even after controlling for the effect of flower position on seed set (Table 4). While differences in seed set between open and supplemented flowers were low (the PL Index ranged from 0.01 to 0.05, Table 5), the PL Indices reported here are similar to estimates of PL obtained in other *xantiana* populations. Moeller et al. (2012) report PL Indices ranging from ≈0 to 0.15, with PL increasing from the western portion of *xantiana's* geographic range toward its eastern range boundary (where it is in contact with *parviflora*). Unlike our findings, which indicate that PL was absent in *xantiana* in 2009, Moeller et al. (2012) found no evidence of interannual variation (2006–2008) in PL in this taxon. In 2009, the region studied here was warmer and received less rainfall than in 2010 (Dudley et al. 2015), which was characterized by high floral densities throughout the Kern River Canyon (Hove and Mazer, personal observation). Given the positive association between precipitation and flower production in *xantiana* (Eckhart et al. 2010), it is plausible that in 2009 floral resources for pollinating insects were scarce, resulting in conditions where seed set was not limited by insufficient pollination.

Other studies of *xantiana* have reported that geographic variation in the occurrence of PL is associated with the composition of pollinator communities and the presence of congener species (Moeller 2005, 2006; Geber and Moeller 2006). Moeller (2004) compared PL among 20 sites in the southern Sierra Nevada that differed in population size and in the presence of pollinator‐sharing congeners. He found that low levels of PL were associated with the presence of at least two flowering congeners and relatively large population sizes. This suggests that a large floral resource in combination with intra‐ and interspecific pollinator sharing enhances pollination service in *xantiana* populations.

In *unguiculata,* no significant differences between the seed set of open and pollen‐supplemented flowers were detected (Table 3). Values of the PL Index were low, differing significantly from zero only in Early 2010 when PL led to a 4.8% reduction in the seed set of open vs. supplemented flowers (PL Index = 0.048, Table 5). The presence of congeners in *unguiculata* populations studied here (Table S1) may have contributed to the absence of PL in this species. Each study population contained at least two congeneric species. *Unguiculata* has a much broader geographic distribution than *xantiana* (Lewis and Lewis 1955), and it is possible that reliability in its interactions with pollinators contributes to this species' success. This is the first published study of PL in natural populations of *unguiculata*; further investigations of PL in populations that vary in size and in co‐occurrence with congeners throughout its range are necessary to corroborate this hypothesis.

Our estimates of self‐pollen deposition (2009) and of self‐fertilization (2008) indicate that the majority of seed production is accomplished through outcrossing in both taxa. Outcrossing rates were high throughout the season in 2008 [mean multilocus outcrossing rates (*t*_m_) ranged from 0.80 to 0.94 in *unguiculata* and 0.76 to 0.95 in *xantiana*] and did not differ between Early‐ and Late‐flowering individuals in either taxon. Based on these estimates, 5–24% of seeds produced in *xantiana* populations could be derived from autogamous or geitonogamous selfing. Our findings, however, suggest that plants receive enough outcross pollen to achieve full seed set. Pollen deposition was similar between emasculated plants (EE treatment) and control plants (II, all flowers intact), and the average number of pollen grains received by emasculated plants exceeded the number of ovules available for fertilization [*unguiculata* mean pollen load (SE) = 251.79 (21.04), *xantiana* mean pollen load (SE) = 592.19 (53.19)]. It is possible, however, that much of this pollen was deposited after stigmas became unreceptive (thereby accounting for the observation that mean seed set was <100%).

In habitats characterized by unpredictable pollination or where the length of the growing season is determined by seasonal events, such as the timing of snowmelt or late‐spring drought, engaging in a mixed‐mating strategy that minimizes dependence on pollinators may help to mitigate the effects of early‐ or late‐season PL (Kalisz and Vogler 2003; Forrest and Thomson 2008, 2010). For example, the timing of snowmelt within populations influences the flowering phenology of the alpine shrub *Phyllodoce aleutica* (Ericaceae). Within populations, poor early‐season pollinator service contributed to both elevated PL and selfing rates among early‐flowering plants (Kameyama and Kudo 2009). Yin et al. (2015) also evaluated day‐to‐day variation in pollinator service, mating system, and seed production in *Incarvillia sinensis* (Bignoniaceae), an annual herb found in the semi‐arid deserts in Inner Mongolia, China. They found that pollinator activity was highly variable (due to windy conditions) and noted a decline in fruit set and seed set, and a corresponding increase in selfing across the flowering season (mean multilocus outcrossing rates (*t*_m_) < 0.40 in the latter half of the flower season). Their findings suggest that mixed mating (accomplished through delayed selfing) may be adaptive when pollinators are scarce or unreliable. While the temporal decline in seed set observed by Yin et al. (2015) is similar to our observations in *unguiculata* and *xantiana*, we found no indication that selfing provides reproductive assurance in these taxa. Seed set in *xantiana* populations was pollen‐limited in 2008, but supplemental pollination increased seed set by <5% (Fig. 3) and the majority of seed production (>70%) was accomplished through outcrossing in that year (Fig. 4). Moreover, pollen receipt in both taxa is accomplished primarily through cross‐pollination; flowers on emasculated and intact plants had similar pollen receipt in 2009.

Outcrossing rates were similar during the Early and Late sampling periods, suggesting that any seasonal decline in pollen quality was not due to an increased tendency toward selfing (Fig. 4). We cannot rule out the possibility that other factors, such as a seasonal decline in pollen quality, independent of the proportion of self‐pollen, contributed to the temporal decline in seed set in these taxa. Pollen viability, pollen germination rates, and pollen tube growth rates are sensitive to increases in temperature (reviewed in Hedhly et al. 2009). The contribution of poor pollen performance to low seed set in these populations remains unknown. In another study, we report pollen germination rates following hand pollinations from individual pollen donors in natural *unguiculata* and *xantiana* populations in 2008. Pollen germination rates (measured as the percent of sampled styles in which at least one pollen tube was observed 2.5 h postpollination) were high in both taxa (84% in *unguiculata* and 94% in *xantiana*) (Hove and Mazer 2013). Pollen attrition rates (measured as the percent of pollen grains deposited on flower stigmas that fail to reach the style base), in natural populations of these taxa, however, are also high (≥65%) (Mazer et al. 2016). Further studies investigating seasonal variation in pollen performance under field conditions are necessary to determine the extent to which environmentally mediated changes in pollen quality affects seed set.

Consistent with the hypothesis that seed set is limited by seasonal ontogenetic or abiotic declines in resource availability, early flowering was associated with elevated seed set at the per‐flower level (independent of the flower position in 2009 and 2010) in the populations sampled here (with the exception of *xantiana* in 2010). If this temporal decline in reproductive performance resulted from a parallel decline in abiotic resources, then an accelerated life cycle – which includes early flowering or short floral life spans – may be favored by natural selection (Guerrant 1989; Sherrard and Maherali 2006; Franks et al. 2007; Aarssen 2008). Previous field‐based estimates of phenotypic selection on an index of flowering progression in these taxa indicate that accelerated reproduction is positively associated with increased whole‐plant fitness in *xantiana* (Hove, Dudley, and Mazer, *unpublished data*), but not *unguiculata* (Dudley et al. 2012). The strength and magnitude of selection favoring early flowering in these taxa is not known. Comparisons of whole‐plant fitness between early‐ and late‐flowering individuals and measurements of selection on flowering time in field populations would be necessary to evaluate whether the temporal declines in seed set reported here may promote the evolution of early or compressed reproduction in these populations.

## Conclusion

We assessed the independent roles of PL, pollen quality, and seasonal declines in resource availability in determining seed set in two late‐flowering annual wildflower taxa endemic to the California Floristic Province, a region whose flora is predicted to be profoundly affected by climate change (Loarie et al. 2008). In the *Clarkia* populations studied here, pollen receipt and pollen quality (estimated as the proportion of selfed vs. outcrossed offspring) remained consistent across the growing season, yet seed set per flower declined between early‐ and late‐flowering plants in the three years of this study. Our results suggest that the resources necessary to provision seeds and/or abiotic conditions decline over the season, reducing the fitness benefits associated with resource allocation to ovules. Whether the temporal reductions in seed set observed indicate that early flowering or accelerated reproduction are adaptive in these populations depends on the genetic basis of flowering time under field conditions in these taxa and the effects of seasonal resource declines on whole‐plant fitness. An exploration of these factors merits further investigation.

## Conflict of Interest

None declared.

## Data Accessibility

Data used in these analyses has been deposited in the Dryad Digital Repository (doi:10.5061/dryad.f68th).

## Supporting information


**Table S1.** Site location and sampling information for the pollen limitation study.
**Table S2.** Site location and sampling information for the floral emasculation study.
**Figure S1.** Relationships between flower position and seed set in *unguiculata* and *xantiana*.
