# Supplementary material for: Seed set variation in wild Clarkia populations: teasing apart the effects of seasonal resource depletion, pollen quality, and pollen quantity
Source: Ecol Evol. 2016 Aug 18;6(18):6524–36. doi: 10.1002/ece3.2372 (PMC5058524; doi:10.1002/ece3.2372)
Supplement: Supplementary file 1 — Table S1. Site location and sampling information for the pollen limitation study. Table S2. Site location and sampling information for the floral emasculation study. Figure S1. Relationships between flower position and seed set in unguiculata and xantiana. [file ECE3-6-6524-s001.doc]

**Table S1**. Populations used for the pollen limitation study in 2008, 2009, and 2010 and their corresponding GPS coordinates, elevations, and sampling dates.

| Year | Taxon | Population Name | Elevation (m) | GPS coordinates | Sampling Dates |
| --- | --- | --- | --- | --- | --- |
| 2008 | *unguiculata* | Mill Creek | 746 | 35**°** 32.24’  118° 36.84’ | 12 May, 17 May |
|  |  | Live Oak | 430 | 35**°** 28.81’  118° 44.89’ | 9 May, 16 May |
|  |  | Stark Creek | 457 | 35**°** 28.44’  118° 43.53’ | 10 May, 16 May |
|  | *xantiana* | Borel Road | 707 | 35**°** 35.04’  118° 31.30’ | 20 May, 1 Jun |
|  |  | Camp 3 | 896 | 35**°** 48.69’  118° 27.70’ | 17 May, 4 Jun |
|  |  | Sawmill Road 1.0 | 932 | 35**°** 40.75’  118**°** 28.45’ | 17 May, 1 Jun |
| 2009 | *unguiculata* | Live Oak | 430 | 35**°** 28.81’  118° 44.89’ | 28 Apr, 7 May |
|  |  | Stark Creek | 457 | 35**°** 28.44’  118° 43.53’ | 6 May, 20 May |
|  | *xantiana* | Borel Road | 707 | 35**°** 35.04’  118° 31.30’ | 21 May, 27 May |
|  |  | Camp 3 | 896 | 35**°** 48.69’  118° 27.70’ | 21 May |
|  |  | Lucas Creek | 510 | 35° 29.038’  118° 42.683’ | 25 Apr, 18 May |
|  |  | Sawmill Road 3.3 | 1336 | 35° 40.48’  118° 30.60’ | 24 May, 8 Jun |
| 2010 | *unguiculata* | Live Oak | 430 | 35**°** 28.81’  118° 44.89’ | 14 May, 3 Jun |
|  |  | Stark Creek | 457 | 35**°** 28.44’  118° 43.53’ | 16 May, 22 May |
|  |  | Mill Creek | 746 | 35**°** 32.24’  118° 36.84’ | 19 May, 3 Jun |
|  | *xantiana* | Borel Road | 707 | 35**°** 35.04’  118° 31.30’ | 5 Jun, 10 Jun |
|  |  | Camp 3 | 896 | 35**°** 48.69’  118° 27.70’ | 2 Jun, 11 Jun |
|  |  | Sawmill Road 3.3 | 1336 | 35° 40.48’  118° 30.60’ | 11 Jun, 25 Jun |

| Taxon | Population Name | Other *Clarkia* taxa noted at site between 2008-2010 |
| --- | --- | --- |
| *unguiculata* | Mill Creek | *C. cylindrica, C. speciosa, C. xantiana* ssp. *xantiana* |
|  | Live Oak | *C. cylindrica,, C. xantiana* ssp. *xantiana* |
|  | Stark Creek | *C. cylindrica, C. exilis* |
| *xantiana* | Borel Road | *C. xantiana* ssp. *parviflora* |
|  | Camp 3 | *C. xantiana* ssp. *parviflora* |
|  | Lucas Creek | *C. cylindrica* |
|  | Sawmill Road 1.0 | None |
|  | Sawmill Road 3.3 | None |

**Table S2**. Populations used for the 2009 floral emasculation study. The first date indicates when the treatments were imposed; the second date is the date of stigma collection.

| Taxon | Population Name | Sampling Dates | # stigmas harvested |
| --- | --- | --- | --- |
| *unguiculata* | Democrat Hot Spring | 19 May  20 May | 91 |
|  | Live Oak | 6 May  8 May | 82 |
|  | Stark Creek | 4 May  7 May | 77 |
| *xantiana* | Greenhorn Mountain Rd. | 8 June  10 June | 83 |
|  | Lucas Creek | 17 May  19 May | 64 |
|  | Sawmill Road 3.3 | 23 May  25 May | 99 |


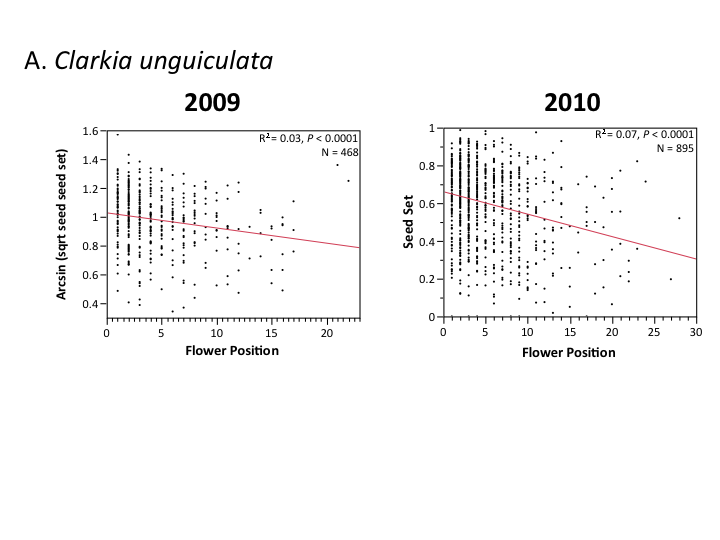


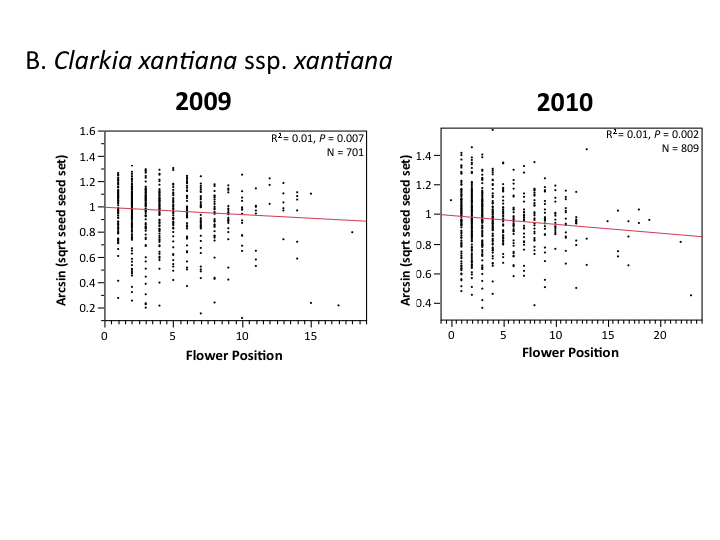


**Figure S1**. 2009 and 2010 relationships between flower position (node number along the primary stem, with the first flower produced defined as being at node “one” and seed set in **A.** *unguiculata* and **B.** *xantiana.* The *Clarkia* taxa studied here produce indeterminate spike-like inflorescences. Flowers at low positions therefore bloom earlier in the season than flowers produced at high inflorescence positions.
